# The miR-876-5p/SOCS4/STAT3 pathway induced the expression of PD-L1 and suppressed antitumor immune responses

**DOI:** 10.1186/s12935-025-03704-2

**Published:** 2025-03-26

**Authors:** Hsuan-Yu Peng, Yu-Li Huang, Ping-Hsiu Wu, Li-Jie Li, Bou-Yue Peng, Chia-Yu Wu, Yu-Lung Lin, Michael Hsiao, Jang-Yang Chang, Peter Mu-Hsin Chang, Hsin-Lun Lee, Wei-Min Chang

**Affiliations:** 1 School of Oral Hygiene, College of Oral Medicine, Taipei Medical University, Taipei, Taiwan; 2 Research Center of Oral Translational Medicine, College of Oral Medicine, Taipei Medical University, Taipei, Taiwan; 3 TMU Research Center of Cancer Translational Medicine, Taipei Medical University, Taipei, Taiwan; 4 Division of Oral and Maxillofacial Surgery, Department of Dentistry, Taipei Medical University Hospital, Taipei, Taiwan; 5 Department of Radiology, School of Medicine, College of Medicine, Taipei Medical University, Taipei, Taiwan; 6 Graduate Institute of Clinical Medicine, College of Medicine, Taipei Medical University, Taipei, Taiwan; 7 Department of Radiation Oncology, Taipei Medical University Hospital, Taipei, Taiwan; 8 TMU Proton Center, Taipei Medical University, Taipei, Taiwan; 9 Program of School of Dentistry, College of Oral Medicine, Taipei Medical University, Taipei, Taiwan; 10 Department of Oral Pathology, Graduate School of Dentistry, Osaka University, Osaka, Japan; 11 School of Dental Technology, College of Oral Medicine, Taipei Medical University, Taipei, Taiwan; 12 Program for Translational Medicine, College of Medical Sciences and Technology, Taipei Medical University, Taipei, Taiwan; 13 Genomics Research Center, Academia Sinica, Taipei, Taiwan; 14 Department of Oncology, Taipei Veterans General Hospital, Taipei, Taiwan; 15 Faculty of Medicine, College of Medicine, National Yang-Ming University, Taipei, Taiwan; 16 Institute of Biopharmaceutical Sciences, National Yang Ming Chiao Tung University, Taipei, Taiwan

**Keywords:** OSCC, MicroRNA, STAT3, SOCS4, Immune evasion

## Abstract

Oral squamous cell carcinoma (OSCC) remains a formidable challenge due to its high recurrence rates and poor prognosis. This study focuses on miR-876, a microRNA significantly associated with OSCC recurrence and clinical outcomes. Analysis of miRNA expression profiles from recurrent OSCC patients revealed that miR-876-5p is markedly upregulated in recurrent tumor tissues and the high expression of miR-876-5p correlates with reduced disease-free and overall survival. Functional assays demonstrated that miR-876 enhances OSCC cell growth, migration, and stemness, contributing to chemoresistance. Mechanistically, miR-876-5p directly targets SOCS4, leading to increased STAT3 activation and subsequent upregulation of PD-L1, which facilitates immune evasion. Additionally, exposure to the tobacco-specific carcinogen NNK was found to induce miR-876 expression and STAT3 activation, implicating environmental factors in miR-876 regulation and promote cancer recurrent. These findings identify the miR-876-5p-SOCS4-STAT3 axis as a critical pathway in OSCC progression, highlighting miR-876-5p as a potential biomarker and therapeutic target to improve treatment outcomes in OSCC patients.

**Supplementary Information:**

The online version contains supplementary material available at 10.1186/s12935-025-03704-2.

## Background

Oral squamous cell carcinoma (OSCC) is one of the most lethal cancers among males in both Taiwan and the USA. Each year, OSCC is responsible for nearly three thousand deaths in Taiwan and approximately ten thousand deaths in the United States [1]. On average, 14.9 patients die daily in Taiwan, and 24 in the USA due to oral cancer [2]. OSCC primarily affects individuals aged 40 to 50, who are often the primary earners for their families. In Taiwan, the direct medical costs associated with OSCC amount to approximately 126 million dollars, with an estimated productivity loss of 342 million US dollars [3]. Smoking is the most significant carcinogen contributing to oral cancer tumorigenesis and malignancy progression [4]. OSCC not only severely impacts public health but also has huge socioeconomic repercussions. The standard treatment for OSCC remains surgery, radiotherapy, and chemotherapy [5]. Unfortunately, OSCC patients often suffer from local functional defects, dysfunctions, malformations, and various intolerable side effects following standard treatments. Additionally, OSCC tumors are prone to metastasis and recurrence, further diminishing patients’ quality of life [6, 7]. Despite advancements in cancer treatments and management that have improved prognoses in many other cancers, the prognosis and treatment outcomes for OSCC patients have shown little improvement.

The survival rate for OSCC remains poor due to frequent late-stage diagnoses and high recurrence rates. The 5-year recurrence rate of OSCC is approximately 50%, with most cases recurring within the first two years after treatment [8]. The current post-treatment standard of care involves ‘watchful waiting’; however, relying solely on clinical exams to detect recurrent disease is highly challenging, as it often depends on patients presenting with new symptoms, such as abnormal bleeding or pain. Recurrent cancer is due to cancer stem cells, which have low immune elimination capacity and high metastasis potential [9]. Consequently, about 90% of recurrent cases have already metastasized by the time they are diagnosed, severely limiting treatment options. As a result, the 5-year overall survival rate for patients with recurrent oral tumors is approximately 30%. Early detection of recurrent or primary lesions—when the tumor is smaller and has not yet spread—is crucial for improving treatment outcomes, as surgery and radiation may still be viable options.

MicroRNAs are small non-coding RNAs that play a critical regulatory role through post-transcriptional mechanisms in both normal physiological functions and disease progression [10]. While microRNAs typically fine-tune gene regulation, their involvement in the competing endogenous RNA network—which coordinates or competes for gene expression among coding genes, long non-coding RNAs, and other microRNAs—highlights their importance as regulatory hubs [11, 12]. MicroRNAs also act as stress responders during cancer treatment [13]. Additionally, microRNAs exhibit high extracellular stability and are easily amplified and detected, making them suitable candidates for liquid biopsy [14, 15]. Several microRNAs have already been utilized as predictive or prognostic biomarkers in cancer, including during recurrence. In this study, we identified a novel miR-876/SOCS4/STAT3/PD-L1 regulatory axis in oral cancer, which promotes cancer stemness and progression, ultimately leading to recurrence and poor prognosis.

## Methods

### Cell lines and culture conditions

Human oral cancer cell lines are listed in the supplementary table S1. All cell culture conditions followed the standard instruction manual from the original cell bank. All media were supplemented with 10% fetal bovine serum, 1% penicillin-streptomycin (PS), and 1% nonessential amino acids (Gibco). Cells were cultured at 37 °C in a 5% CO₂ atmosphere and maintained within 3 months of resuscitation from frozen aliquots, with fewer than 20 passages used for each experiment. Additionally, all cells were regularly checked for mycoplasma infection and cell morphology to ensure they remained healthy.

### CCK-8 assay

OSCC cells were transfected with miR-876 mimics or a non-targeting control (N.C.) plated in a 96-well plate and cultured for an additional 72 h. Cell growth was assessed using the Cell Counting Kit-8 (CCK-8) (Cayman Chemical, Ann Arbor, MI). Ten microliters of CCK-8 solution were added to each well, followed by incubation for 3 h. Absorbance at 450 nm was measured using a Thermo Scientific Multiskan FC Microplate Photometer.

### Transwell migration assays

Transwell migration assays were performed using a Transwell insert plate (PET membrane, 8-µm pore size, NEST, Wuxi, China) without coating. For the Cal-27 cell migration assay, Cal-27 cells (3 × 10^4^ cells per well) were seeded in the upper chamber in serum‐free medium, and 600 µL of medium containing 2% FBS was added to the lower well. Cells were plated in the top chamber and incubated for 24 h to allow them to attach. After 24 h of incubation, migrated cells were fixed on the membrane with 70% ethanol for 60 min at 4°C and washed twice with PBS. The interiors of the inserts were then cleaned with wet cotton swabs. Cells were stained with a cell stain solution (0.1% crystal violet, 20% methanol) and visualized under an inverted microscope (200× magnification; Zeiss Axiovert 200, Carl Zeiss, Oberkochen, Germany). Images were then counted using an analytical imaging station software package (Imaging Research, Ontario, Canada).

### Spheroid formation analysis

For spheroid formation analysis, 1000 OSCC cells were co-cultured in a 24-well ultra-low attachment dish in a humidified 37 °C incubator for 7–14 days as our previous condition [16]. The number of spheroids with a green signal and a diameter ≥ 100 μm were counted under fluorescence microscopy (Zeiss Axiovert 200, Carl Zeiss, Oberkochen, Germany).

### RNA extraction and real-time PCR (qRT‐PCR)

Total RNA was extracted from cell lines using TRIzol reagent (Life Technologies, Gaithersburg, MD, USA). cDNA was synthesized using the Maxima First Strand cDNA Synthesis Kit for qRT-PCR (Thermo Fisher Scientific, Carlsbad, CA, USA). Reverse-transcription reactions were performed for NANOG, NOTCH1, Oct4 (POU5F1), ABCG2, PCNA, Ki67 (MKI67), PDL-1, SOX2, and SOCS4 mRNAs, with GAPDH as an internal reference. Primer sequences are listed in Supplementary Table S1. qRT-PCR analysis was conducted using ChamQ SYBR Color qPCR Master Mix (Vazyme, Nanjing, China) with MB-Q96-LP/ MB-QSM System (Gunster Biotech, New Taipei City, Taiwan) on an Applied Biosystems QuantStudio 3 real-time PCR system. Expression levels were calculated as ▵▵Ct after normalization with the reference control. For mature miRNA detection, cDNA was prepared using the miScript II RT Kit (Cat: 218161, QIAGEN, Valencia, CA). Real-time PCR was performed using ChamQ SYBR Color qPCR Master Mix (Vazyme) and analyzed on an Applied Biosystems QuantStudio 3 system. U6 served as the internal control for miRNA detection. Fold changes were calculated using the 2-ΔΔCt method.

### Protein extraction and Western blot analysis

Cells were lysed in RIPA lysis buffer (containing 50 mM Tris-HCl, 1% NP-40, 150 mM NaCl, 0.1% SDS, 1 mM PMSF, 1 mM Na₃VO₄) with a complete protease and protease inhibitor cocktail (1:1000) (Sigma-Aldrich, Inc.). Protein concentrations were then determined using the BCA assay kit (Thermo, USA) following the manufacturer’s instructions. Then, 10–30 µg of protein lysates from cells were loaded onto 10% SDS polyacrylamide gels and subjected to electrophoresis, followed by transferring the proteins to polyvinylidene fluoride membranes (Pall Life Sciences, Glen Cove, NY). The membranes were probed with antibodies. The antibodies information is listed in the supplementary Table S1. Antibodies against GAPDH were used as an internal control. The membranes were exposed using Immobilon ECL Ultra Western HRP Substrate (Merck-Millipore, Burlington, MA, USA) for the HRP-coupled secondary antibodies and were analyzed using the e-BLOT Touch Imager (e-BLOT, Shanghai, China). Protein levels were detected as the integrated area (pixels) of the band intensities using densitometry analysis with ImageJ software (Bethesda, MD, USA). The numerical values for protein band intensities were corrected with the values for the GAPDH or bactin bands. All protein bands were quantified using ImageJ, and the statistical results are listed in Supplementary Table S2.

### Plasmids and transfection

The pLX304-SOCS4 or STAT3 plasmid was generated by inserting full-length cDNA (SOCS4: NM_199421.2, STAT3: NM_139276.3) into the vector. The mature miR-876 and sponge miR-876 were generated by gene synthesis and subclone into pLV-U6-SGIPZ vector (Addgene # 174847) by XhoI and BamHI. miR-876 mimics (PM) (ID: 12319, Cat: AM17100, Thermo Fisher Scientific) transfection mix was prepared in a TransIT-X2 Transfection Reagent (Mirus, Madison, WI, USA), following the manufacturer’s protocol and plated on Cal-27, SCC-4, and SCC-15 cells. For lentivirus production, 293T cells were co-transfected with pMD.G, pCMV▵R8.91, and lentiviral transfer plasmids using TransIT-LT1 Transfection Reagent (Mirus, Madison, WI, USA). The culture medium containing lentivirus was harvested 48 h after transfection. Cells were removed from the culture medium by centrifugation at 500 g for 5 min and filtered through a 0.45-µm filter. The lentiviral stocks in the medium containing 8 µg/mL polybrene were used to infect the target cells for 6 h.

### Luciferase reporter assays

The oncogenic transcriptional factor activity is determined by SBI Signaling Pathway Reporters (Palo Alto, CA) in Cal-27 cells and followed by our previous approach [7]. For 3’UTR luciferase reporter vector of SOCS4 was generated by primer synthesis of different miR-876 response elements of human SOCS4 in a modified pGreenfire CMV vector with CpoI site after the luciferase gene. 293T cells were cultured in 24-well plates and co-transfected with 300 ng of SOCS4 3’-untranslated region (UTR) reporter plasmid, along with 25 nM of miR-875-5p mimics (PM) or non-targeting control (N.C.), using TransIT-X2 Transfection Reagent (Mirus, Madison, WI) according to the manufacturer’s instructions. The luciferase assay was performed 24 h post-transfection with the ONE-Glo™ Luciferase Reporter Assay Substrate (Promega, Madison, WI) following the manufacturer’s protocol. The 3’UTR reporter activity was measured by SpectraMaxID3 reader (Molecular Devices, San Jose, CA).

### Jurkat T-mediated tumor killing assay

Jurkat T cells were activated by stimulation with 1.25 µg/ml of monoclonal anti-CD3 (Clone: OKT3) and 1.25 µg/ml anti-CD28 (Clone: CD28.2) antibody for 48 h. The tumor-killing assay followed our previous protocol [17]. Tumor cells with green fluorescence (2 × 10⁶) were pre-plated in a 6-cm dish. The tumor cells were co-cultured with the activated Jurkat T cells at a tumor cell/T cell ratio of 1:5 for 24 h. After co-culture, all cells were collected using trypsin, labeled with propidium iodide (PI), and subjected to flow cytometry (Thermo Fisher Scientific, Waltham, MA). The PI-positive (PI+) cells from the tumor cell population gated by BL1 + represented dead tumor cells killed by T cells.

### Flow cytometry

The cells were harvested, washed twice with PBS, fixed in 1% paraformaldehyde (PFA) overnight at 4 °C, washed, and resuspended in flow cytometry buffer (1× PBS containing 1% FBS). To determine surface markers, these cells were labeled with the surface fluorochrome-conjugated antibodies CD274 (PD-L1)-APC (#329707), CD45-APC (#368511), or CD69-FITC (#310903) for 60 min on ice in the dark. The stained cells were analyzed using an Attune NxT Flow Cytometer (Thermo Fisher Scientific, Waltham, MA) and the quantitative analysis was performed using FlowJo v10 (Treestar, Ashland, OR).

### Statistical analysis

All experiments were repeated in at least 3 independent experiments. Data were presented as the mean ± standard deviation (SD) from repeated independent experiments. The significant differences between different treatment groups were assessed using the Student’s t-test. Between-group differences were considered significant at *P* < 0.05. Data analyses were performed using GraphPad Prism Ver. 8 (San Diego, CA, USA).

## Results

### miR-876 is significantly associated with oral cancer recurrence and poor prognosis

**Fig. 1:**
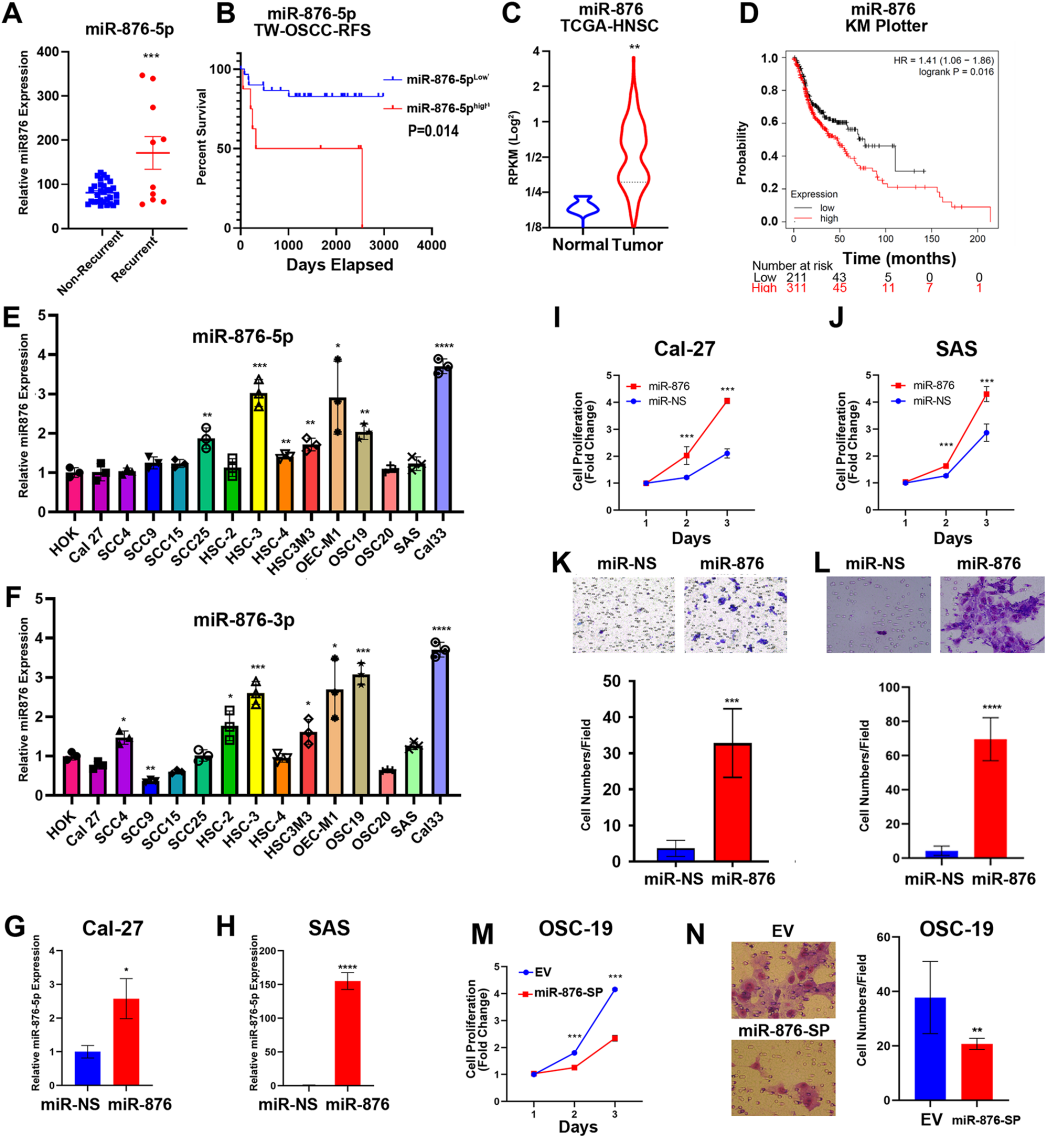
miR-876 is Significantly Associated with Oral Cancer Recurrence and Poor Prognosis **(A)** Differential expression of miR-876-5p between recurrent and non-recurrent OSCC patients from the GSE45238 dataset. **(B)** Kaplan–Meier curves showing relapse-free survival times based on differential miR-876-5p expression in the GSE45238 cohort. **(C)** Analysis of the TCGA HNSCC cohort demonstrating differential miR-876 expression between tumor and normal tissues. **(D)** Kaplan–Meier curves for overall survival, indicating a significant association between miR-876 expression levels and survival outcomes in 522 OSCC samples from the Kaplan-Meier Plotter (https://kmplot.com/analysis/). **E-F.** qRT-PCR analysis validating the expression levels of miR-875-5p/3p in OSCC cell lines and conditioned medium. U6 small nuclear RNA was used as the internal normalization control. G-H. qRT-PCR analysis of miR-876-5p expression in ectopic miR-876-5p expression in Cal-27 (**G**) and SAS (**H**) cells. **I-J.** Relative proliferation of Cal27 **(I)** and SAS **(J)** cells with stable overexpression of miR-876, assessed over 24 to 72 h using the CCK8 assay. Data are represented as mean ± SD; ***, *p* < 0.001 versus vector control. **K-L.** Representative images and quantified bar chart of Transwell migration assays showing migration of Cal27 (K) and SAS (L) cells with stable overexpression of miR-876, assessed over 24 h. **M.** Relative proliferation of miR-876-5p sponge OSC-19 cell. **M.** Transwell migration assays of miR-876-5p sponge OSC-19 cell. All experiments were repeated in at least 3 independent experiments (*n* = 3). Data are presented as mean ± SD; *, *p* < 0.05; **, *p* < 0.01; ***, *p* < 0.001 versus miR-NS control

To identify critical microRNAs involved in oral cancer recurrence, we analyzed the differential expression of miRNAs in recurrent oral cancer patients from Taiwan (GSE45238, Table S3) [18, 19]. miR-876-5p emerged as one of the most significantly altered microRNAs in recurrent oral cancer (Fig. 1A) and high miR-876-5p expression had a shorter recurrence-free survival time (Fig. 1B). Additionally, we further validated miR-876 expression in an independent TCGA-HNSC cohort revealing significant upregulation of miR-876 in head and neck cancer tumors relative to corresponding normal tissues (Fig. 1C, *p* = 0.002). Moreover, we also investigated the relationship between miR-876 expression and clinical outcomes using data from the Kaplan-Meier plotter database on head and neck cancer patients [20]. The group with high miR-876 expression exhibited significantly shorter overall survival (OS) compared to the low-miR-876 group (median OS: 77.3 months vs. 47.67 months, Fig. 1D). We assessed miR-876 levels in 14 OSCC cell lines and human oral keratinocytes (HOK) using qRT-PCR. The results showed that miR-876 expression was elevated in most OSCC cell lines, particularly in OSC-19, Cal-33, OEC-M1, and HSC-3, compared to HOK (Fig. 1E–F). In contrast, Cal-27, SAS, SCC-4, and SCC-15 exhibited expression levels similar to those observed in normal keratinocytes. Interestingly, miR-876 was epigenetically silenced in Cal-27 cells; however, treatment with the DNMT inhibitor 5-Aza-dC restored the expression of both miR-876 and miR-873, which are located in the same chromosomal cluster (Supplementary Fig. S1). To explore the functional role of miR-876 in OSCC progression, we induced ectopic miR-876 expression in Cal-27 and SAS cells (Fig. 1G-H), both of which exhibit relatively low endogenous miR-876 levels similar to those in HOK. miR-876 overexpression promoted in vitro growth (Fig. 1I-J) and migration (Fig. 1K-L) in both cell lines. Moreover, the miR-876-5p sponge vector reduced proliferation (Fig. 1M) and migration (Fig. 1N) in OSC-19 cells, which express high levels of endogenous miR-876. Taken together, these results suggest that miR-876 functions as an oncogenic miRNA contributing to OSCC progression.

### miR-876 enhances Oscc chemoresistance and stemness

**Fig. 2:**
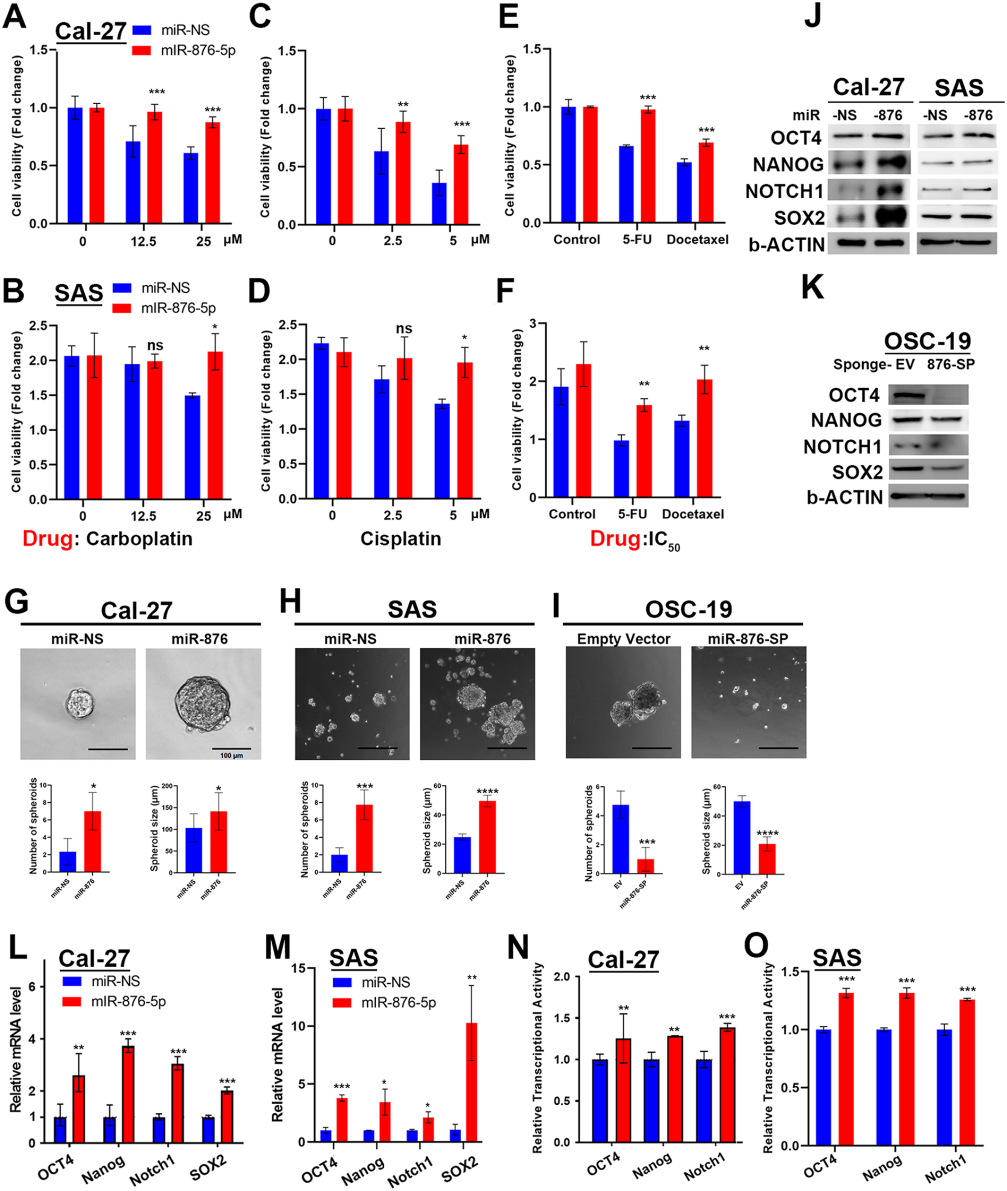
miR-876 Enhances OSCC Chemoresistance and Stemness **A-F.** Chemoresistance assays in OSCC cells overexpressing miR-876. A-D. Cell viability in miR-876-overexpressing Cal27 and SAS cells treated with Carboplatin (12.5 and 25 µM) or Cisplatin (2.5 and 5 µM) for 48 h, assessed using the CCK8 assay. E-F. Cell viability in miR-876-overexpressing Cal27 and SAS cells treated with 5 µM 5-FU and 5 µM Docetaxel for 24 h, assessed using the CCK8 assay. **G-I.** Representative images and quantified bar chart of the sphere formation assay comparing Cal27 (**G**) and SAS (**H**) cells transfected with miR-876 or control vector and OSC-19 expressed miR-876-5p sponge or empty vector (**I**). **J** Western blot analysis of Nanog, SOX2, and Notch1 protein levels after transfection with miR-876-5p mimics (50 nM for 48 h) in Cal27 and SAS cells. **K.** Western blot analysis in miR-876-5p sponge OSC-19 cells. β-Actin was used as an internal control. **L-M.** qPCR analysis of OCT4, Nanog, Notch1, and SOX2 transcripts after miR-876 transfection in Cal27 (**L**) and SAS (**M**) cells for 48 h. GAPDH was used as an internal control. **N-O**. The effect of miR-876 on the transcriptional activity of constructs containing cis-acting elements of OCT4, Nanog, and Notch1 in Cal-27 (**N**) and SAS (**O**) cells, as measured by relative luciferase activity compared to control mimics. **N.** Western blot analysis of Nanog, SOX2, and Notch1 protein levels after transfection with miR-876-5p mimics (50 nM for 48 h) in Cal27 and SAS cells. All experiments were repeated in at least 3 independent experiments (*n* = 3). Data are presented as mean ± SD; *, *p* < 0.05; **, *p* < 0.01; ***, *p* < 0.001 versus miR-NS control

Cancer stemness is crucial for tumor initiation, metastasis, drug resistance, and recurrence. Chemoresistance is a key feature of recurrent oral cancer. Cisplatin, carboplatin, fluorouracil, and docetaxel are commonly used chemotherapy drugs in oral cancer treatment. To investigate the role of miR-876-5p in OSCC recurrence and chemoresistance, we demonstrated that miR-876 overexpression reduced the cytotoxicity induced by anticancer drugs (Fig. 2A–F). In addition, miR-876 enhanced cancer stemness, as evidenced by increased sphere formation in OSCC cells with low endogenous miR-876 expression (Cal-27 and SAS cells; Fig. 2G–H). Conversely, depletion of miR-876-5p function reduced sphere formation in OSC-19 cells (Fig. 2I). We also observed that miR-876 upregulated the expression of key stemness transcription factors—including OCT4, SOX2, Nanog, and Notch1—at both the protein (Fig. 2J) and mRNA levels (Fig. 2L–M), and increased their transcriptional activity (Fig. 2O–P). Moreover, depleting miR-876-5p function using a sponge vector also reduced the expression of these critical cancer stemness transcription factors (Fig. 2K). These results suggest that miR-876 plays a pivotal role in chemoresistance by enhancing cancer stemness in OSCC.

### miR-876-5p directly regulates SOCS4 in OSCC

**Fig. 3:**
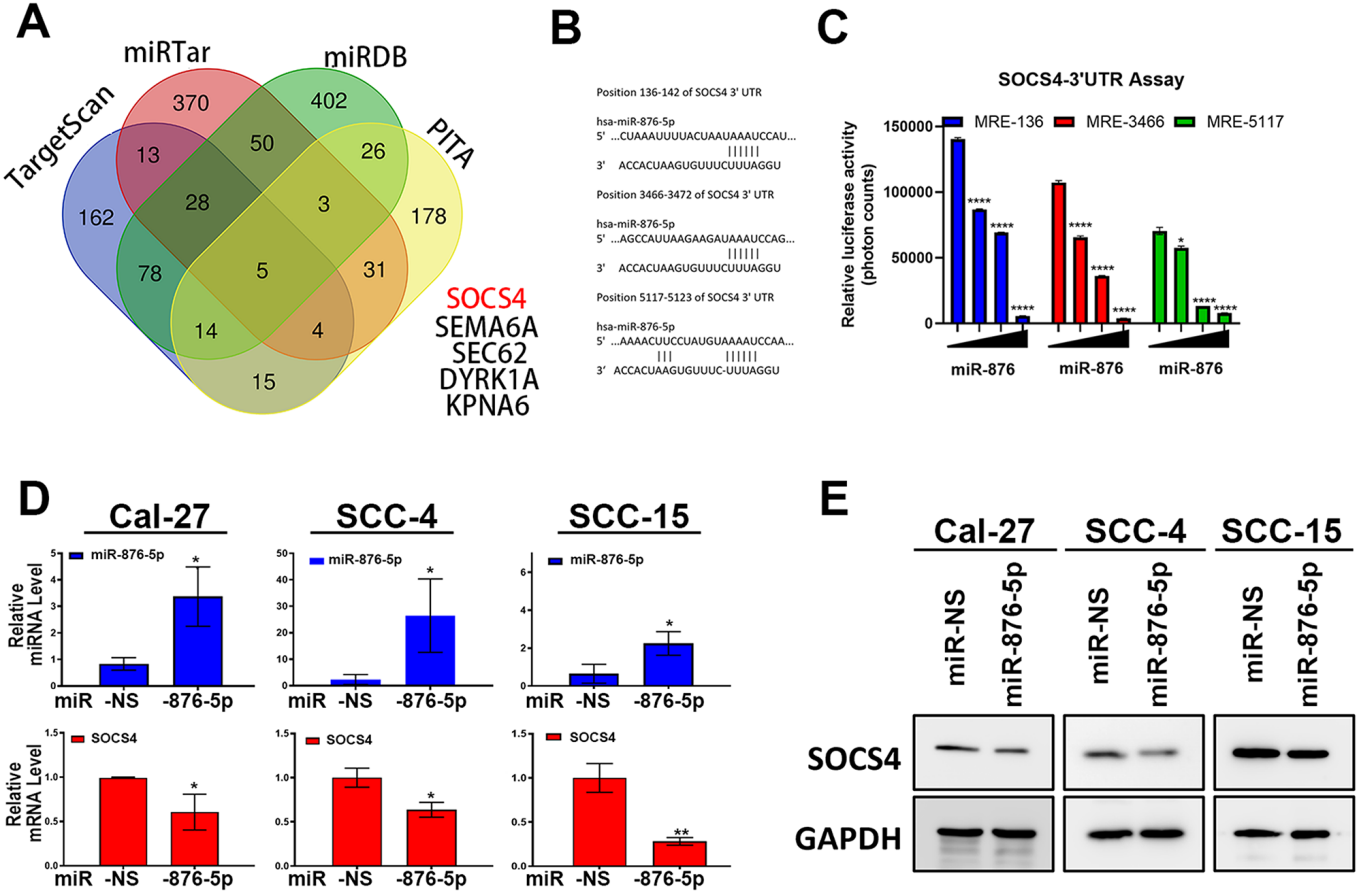
miR-876-5p Directly Regulates SOCS4 in OSCC **(A)** Schematic representation of the putative miR‐876-5p binding sequence in the 3′‐UTR of SOCS4. **(B)** Luciferase activity assay showing the effect of miR-876-5p mimics (PM, 50 nM) on constructs containing the wild-type 3′-UTR of SOCS4 in 293T cells. Relative luciferase activity was measured 48 h post-transfection and normalized to cell number. **(C)** qRT-PCR analysis of miR-876-5p and SOCS4 expression levels in Cal27, SCC-4, and SCC-15 cell lines following transfection with miR-876 mimics (50 nM for 48 h). GAPDH served as internal control. **(D)** Western blot analysis of SOCS4 protein levels following transfection with miR-876-5p mimics (50 nM for 48 h) in Cal27, SCC-4, and SCC-15 cell lines. GAPDH was used as an internal control. Figure 4. The effects of dysregulated SOCS4 in STAT3 activity and stem cell markers. All experiments were repeated in at least 3 independent experiments (*n* = 3). Data are presented as mean ± SD; *, *p* < 0.05; **, *p* < 0.01; ****, *p* < 0.0001 versus control

**Fig. 4:**
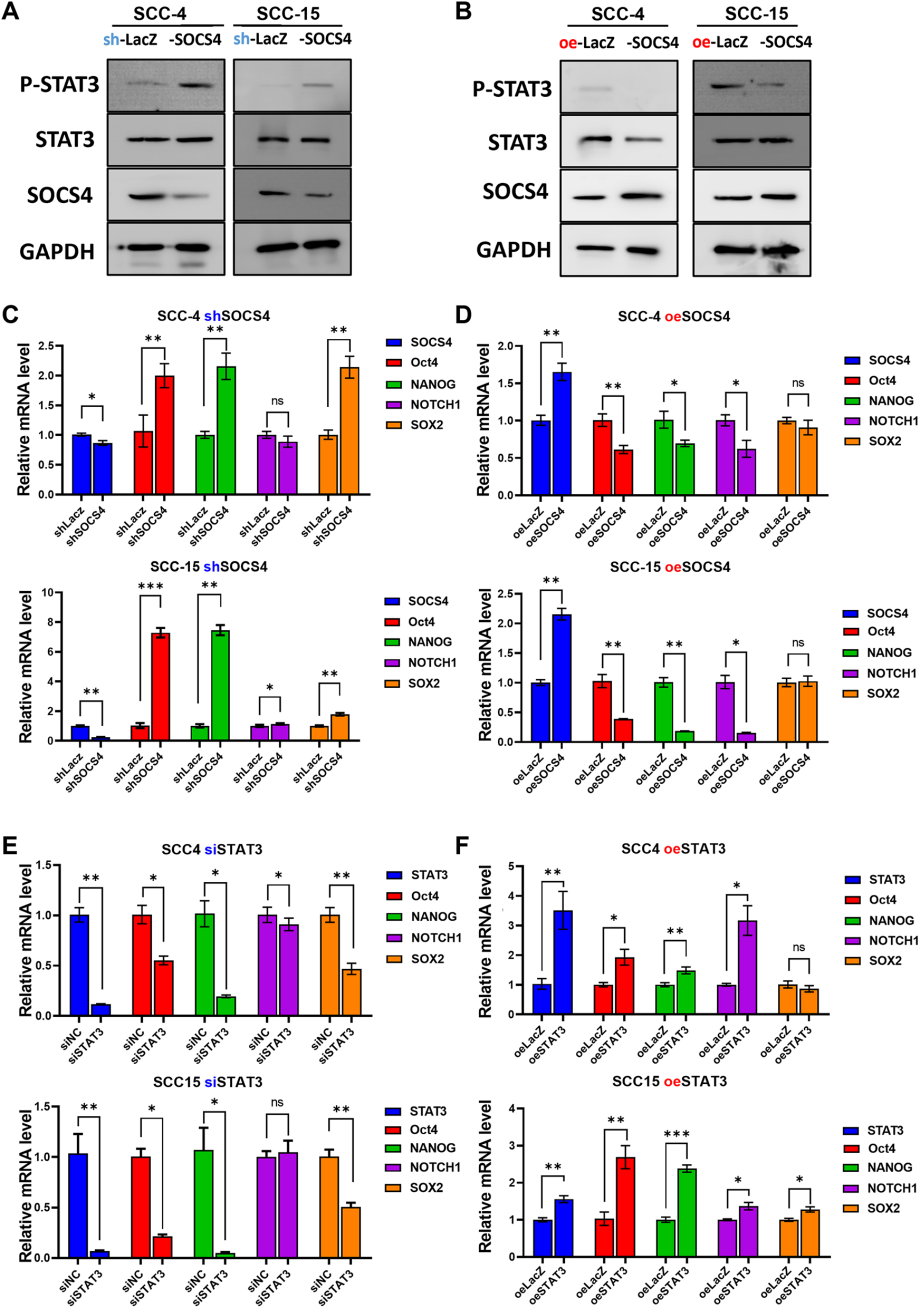
Impact of SOCS4 Modulation on STAT3 Signaling and Stemness Markers in OSCC Cells **A**. Western blot analysis of STAT3 and phospho-STAT3 levels in SCC-4 and SCC-15 cells infected with lentivirus expressing SOCS4 shRNA or non-targeting shRNA (Lacz) for 48 h. **B**. Western blot analysis of STAT3 and phospho-STAT3 levels in SCC-4 and SCC-15 cells following lentiviral SOCS4 overexpression for 48 h. GAPDH was the loading control. **C-D**. qRT-PCR analysis of SOCS4, OCT4, Nanog, Notch1, and SOX2 transcript levels following SOCS4 knockdown (**C**) or overexpression (**D**) in SCC-4 and SCC-15 cells. **E-F.** qRT-PCR analysis of SOCS4, OCT4, Nanog, Notch1, and SOX2 transcript levels following transient SOCS4 silencing (**E**) or STAT3 overexpression (**F**) in SCC-4 and SCC-15 cells. GAPDH was the internal control. All experiments were repeated in at least 3 independent experiments (*n* = 3). Data are presented as mean ± SD; *, *p* < 0.05; **, *p* < 0.01; ***, *p* < 0.001 versus control

To identify the direct targets of miR-876-5p in oral cancer recurrence, we used the TargetScan database, which predicts miRNA targets based on binding free energy and species conservation. To find the potential miR-876-5p target, we used TargetScan [21], miRDB [22], and miRTarbase [23], and PITA [24] and identified five potential targets: SOCS4, SEMA6A, Sect. 62, DYRLA, and KPNA6 (Fig. 3A). SOCS4 is a critical regulator of STAT signaling and plays an important role in immune-related cancers [25]. Computational screening identified SOCS4 as a potential target, with multiple miR-876 binding elements (MREs) in the SOCS4 3’-UTR (Fig. 3C). To confirm whether miR-876 directly regulates SOCS4 expression, we conducted 3’-UTR luciferase reporter assays, which showed that miR‐876-5p mimic (PM) transfection significantly suppressed SOCS4 3’-UTR luciferase activity (Fig. 3B). Additionally, miR‐876-5p mimics significantly reduced SOCS4 mRNA and protein levels (Fig. 3C-D). We further examined the impact of SOCS4 on STAT3 activation by silencing SOCS4 with shRNA, which resulted in increased STAT3 phosphorylation (Fig. 4A). Conversely, SOCS4 overexpression led to decreased STAT3 phosphorylation (Fig. 4B). Furthermore, the expression of stemness transcription factors, such as Oct4, Nanog, Notch1, and Sox2, was inversely correlated with SOCS4 expression (Fig. 4C-D). The expression of these transcription factors was also significantly altered by modulating STAT3 expression (Fig. 4E-F). These findings suggest that SOCS4 overexpression suppresses STAT3 activity and downstream stemness transcription factors in OSCC cells.

### miR-876-5p promotes OSCC stemness through direct Inhibition of SOCS4

**Fig. 5:**
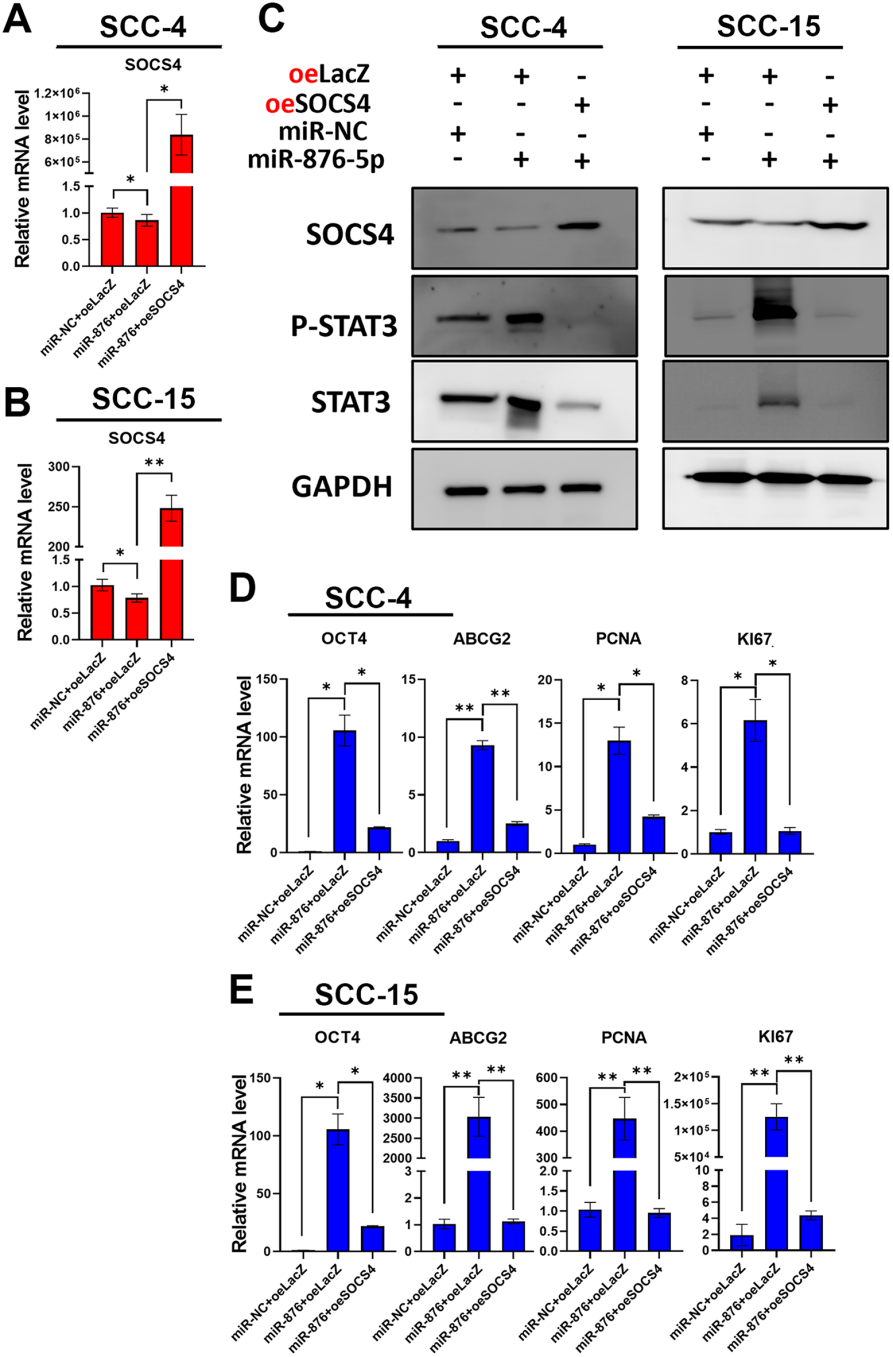
Impact of miR-876-5p and SOCS4 Co-transfection on STAT3 Signaling and Gene Expression in OSCC Cells **A-B.** qPCR analysis of SOCS4 expression in SCC-4 **(A)** and SCC-15 **(B)** cells co-transfected with miR-876-5p mimics or non-targeting control (NC) and a SOCS4 expression vector (lacking 3’-UTR) or control vector for 48 h. **C.** Western blot analysis of SOCS4, STAT3, and phospho-STAT3 in SCC-4 and SCC-15 cells. GAPDH was used as the protein loading control. **D-E.** qPCR analysis of cancer stemness markers (OCT4, ABCG2) and proliferation genes (PCNA, KI67) in SCC-4 **(D)** and SCC-15 **(E)** cells. GAPDH was used as the internal control. All experiments were repeated in at least 3 independent experiments (*n* = 3). Data are presented as mean ± SD; *, *p* < 0.05; **, *p* < 0.01 versus control

To investigate whether miR-876-5p-induced downregulation of SOCS4 plays a critical role in OSCC stemness and recurrence via STAT3, we generated a SOCS4 expression vector lacking the 3’-UTR (SOCS4-CDS), which is not targeted by miR-876-5p and restores SOCS4 expression and function (Fig. 5A-B). In OSCC cells, SOCS4 restoration abolished the miR-876-5p-induced increase in STAT3 phosphorylation (Fig. 5C). Moreover, SOCS4 restoration regulated the expression of downstream cancer stemness and proliferation genes (Fig. 5D-E). These results indicate that the miR-876-5p-SOCS4-STAT3 axis is a critical regulatory pathway in OSCC stemness, proliferation, and recurrence.

### miR-876 upregulates PD-L1 expression induced by STAT3

**Fig. 6:**
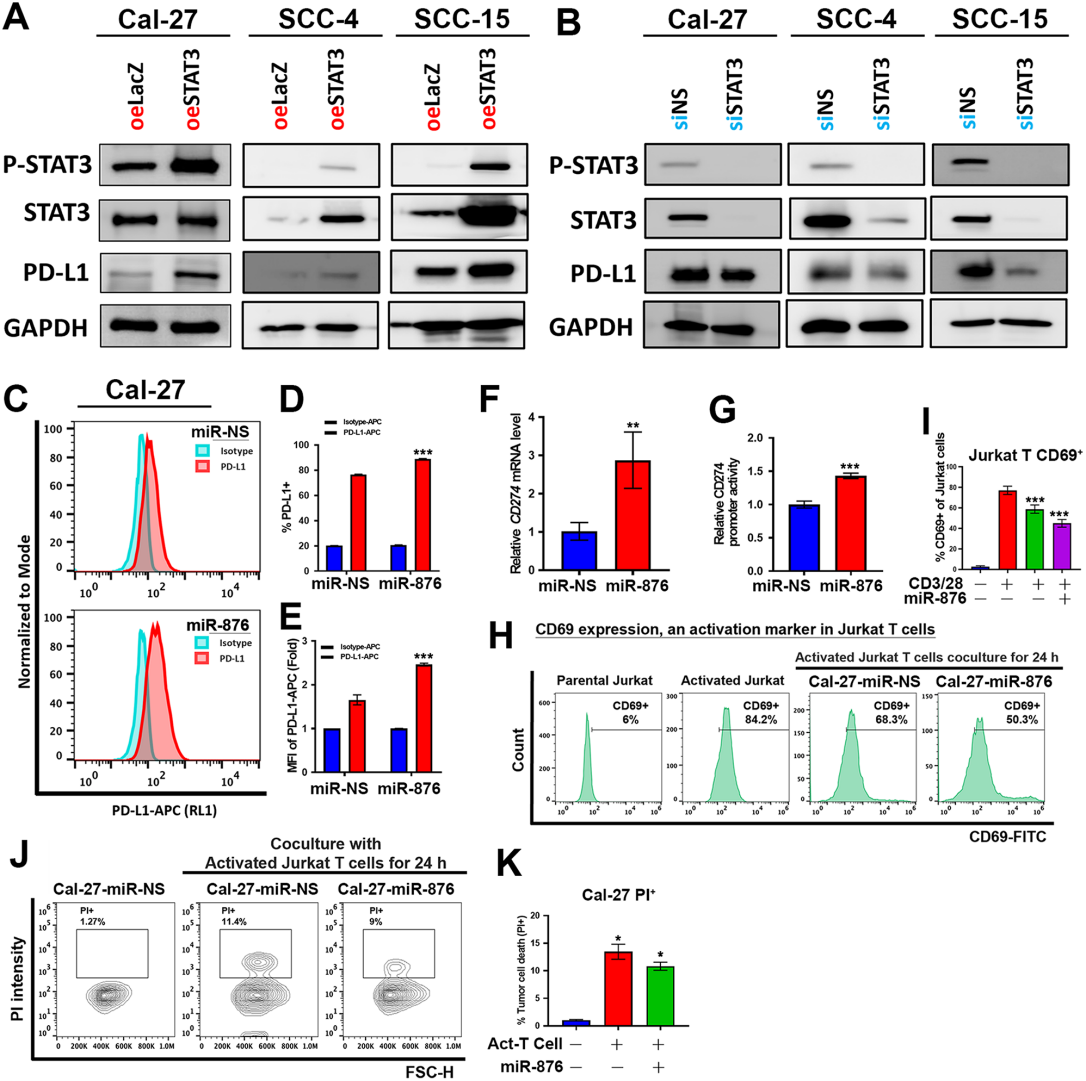
The miR-876/SOCS4/STAT3 Pathway Induces PD-L1 Expression and Suppresses Antitumor Immune Responses. **A-B.** Western blot analysis of PD-L1, STAT3, and phospho-STAT3 in Cal27, SCC-4, and SCC-15 cells infected with STAT3-expressing lentivirus (**A**) or transfected with siRNA-STAT3 **(B)** for 48 h. GAPDH was the loading control. **C-E.** Flow cytometry analysis of PD-L1 + cells in Cal27 cells following transfection with miR-876 or miR-control mimics. (**C**) shows representative plots, (**D)** quantifies percentage of PD-L1 + cells, and (E) compares mean fluorescence intensity of PD-L1 in OSCC cells. **F.** qPCR analysis of PD-L1 expression in Cal27 cells after miR-876 or miR-control mimic transfection, normalized to GAPDH. **G.** Luciferase assay measuring the effect of miR-876 on PD-L1 promoter activity, relative to Renilla luciferase and miR-control mimics. All experiments were repeated in at least 3 independent experiments (*n* = 3). Data are presented as mean ± SD; *, *p* < 0.05; **, *p* < 0.01; ***, *p* < 0.001 versus miR-NS control. **H-K**. Jurkat T cell activation **(H)** and tumor cell killing **(J)** after co-culture with Cal27 cells transfected with miR-876 or miR-control mimics. CD69 expression (active T cells, **I**) and PI staining (dead cancer cells, **K**) were assessed by flow cytometry

Recent studies suggest that recurrent OSCC exhibits a cancer immune microenvironment characterized by immune evasion, with PD-L1 being a key immune checkpoint differentially expressed in recurrent cancer cells [26]. STAT3 is a potent inducer of PD-L1 expression in oral cancers [27] to explore whether STAT3 promotes immune evasion via PD-L1 expression, we transfected OSCC cells with either a STAT3 expression plasmid or STAT3 siRNA. Increased STAT3 expression led to a marked upregulation of PD-L1 expression (Fig. 6A), while STAT3 knockdown resulted in reduced PD-L1 expression (Fig. 6B). In Cal-27 cells with low miR-876 expression, miR-876 transfection induced surface PD-L1 (Fig. 6C-E) and CD274 (PD-L1) mRNA (Fig. 6F) expression. The CD274 promoter activity was also stimulated by miR-876-5p transfection (Fig. 6G). PD-L1 is the major immune checkpoint ligand that suppresses adaptive immune responses [28]. To assess the role of miR-876 in cancer immune evasion, we conducted an in vitro T cell cytotoxicity assay. Jurkat cells were first activated with α-CD3/CD28 beads for three days and then co-cultured with either miR-876 or control miR-transfected Cal-27 cells, respectively. After 48 h, T cell activation and cytotoxicity were evaluated via CD69 staining and intracellular PI accumulation using flow cytometry. We observed that miR-876-expressing OSCC cells induced lower T cell activation (Fig. 6H-I) and reduce T cell-induced antitumor cytotoxicity (Fig. 6J-K) compared to control cells, indicating that miR-876-5p promotes PD-L1-mediated immune evasion and reduces T cell-induced cell death in OSCC.

### miR-876 levels increase in OSCC cells following exposure to NNK

**Fig. 7:**
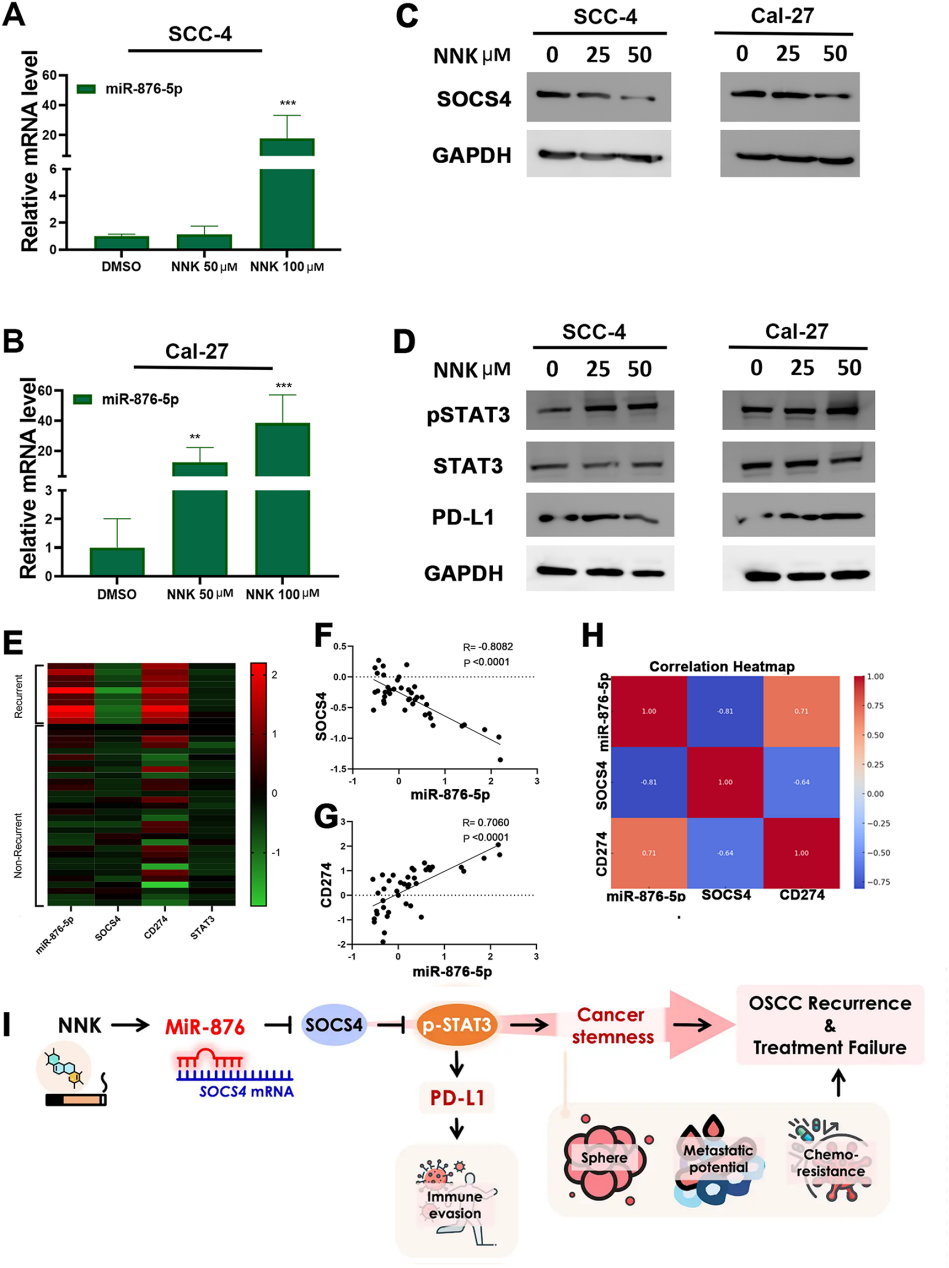
The Effects of NNK on miR-876 Expression and STAT3 Activation **A-B.** miR-876-5p expression level in SCC-4 **(A)** and Cal-27 **(B)** cells were treated with 50 µM NNK for 72 h. **C-D.** Western blot analysis of SOCS4 (C), STAT3, phospho-STAT3, PD-L1 and GAPDH (loading control) in SCC-4 and Cal-27 cells treated with 25–50 µM NNK for 72 h. All experiments were repeated in at least 3 independent experiments (*n* = 3). Data are presented as mean ± SD; **, *p* < 0.01; ***, *p* < 0.001 versus DMSO control **E.** Heatmaps of miR-876-5p, SOCS4, CD274, and STAT3 expression from the Taiwan OSCC cohort (GSE37991 and GSE45238). **F-G**. Pearson correlation analysis between SOCS4/miR-876-5p **(F)** and CD274/miR-876-5p (G) from Taiwan OSCC cohort. H. Correlation heatmap analysis among miR-876-5p, SOCS4, and CD274. **I.** Proposed model: miR-876 mediates SOCS4/STAT3/PD-L1 signaling in OSCC cells, leading to inhibition of T cell activation

Our findings suggest that dysregulation of miR-876 significantly influences OSCC recurrence and progression. However, the molecular mechanisms driving miR-876 expression remain unclear. Recent evidence links cigarette smoke exposure to various diseases, including cancer and inflammation [29]. Cigarette smoke has also been associated with increased STAT3 phosphorylation in several cancer types, including head and neck cancer [19, 30]. Tobacco-specific nitrosamines, such as 4-(Methylnitrosamino)-1-(3-pyridyl)-1-butanone (NNK), are major components of cigarette smoke and potent carcinogens. We hypothesized that NNK may induce miR-876 expression and subsequent STAT3 activation in OSCC recurrence. To test this hypothesis, we treated OSCC cells with different concentrations of NNK and found that NNK significantly stimulated miR-876 expression (Fig. 7A–B) and further suppressed SOCS4 expression (Fig. 7C). Furthermore, NNK promoted STAT3 activation and PD-L1 expression (Fig. 7D) in a concentration-dependent manner. These results demonstrate that NNK exposure significantly induces miR-876 expression and STAT3 activation, suggesting that cigarette smoke critically influences the miR-876–STAT3 axis in OSCC.

To validate the miR-876-5p–SOCS4–PD-L1 (gene symbol: CD274) axis in clinical patients, we further analyzed the expression of these genes in OSCC patients from Taiwan (Fig. 7E, Table S4). We observed a negative correlation between miR-876-5p and SOCS4 (Fig. 7F) and a positive correlation between miR-876-5p and PD-L1 expression (Fig. 7G). The correlation heatmap also revealed the clinical association among the miR-876-5p–SOCS4–PD-L1 axis.

Overall, these findings demonstrate that NNK exposure significantly induces miR-876 expression, suppresses SOCS4, and promotes STAT3 activation, ultimately leading to increased PD-L1 expression. These results suggest that cigarette smoke critically influences the miR-876–STAT3 axis in OSCC.

## Discussion

Our findings identify miR-876-5p as a crucial molecule in OSCC recurrence and poor prognosis (Fig. 7I). Elevated levels of miR-876-5p were associated with shorter overall survival and were significantly upregulated in OSCC tissues and cell lines. Functionally, miR-876 was shown to promote OSCC cell growth, migration, and cancer stemness, thereby contributing to chemoresistance. Mechanistically, miR-876 directly targets SOCS4, activating the STAT3 signaling pathway, which subsequently upregulates PD-L1 expression, facilitating tumor immune evasion. Additionally, exposure to the tobacco-specific carcinogen NNK was found to induce miR-876 expression and STAT3 activation, suggesting that environmental factors may play a role in miR-876 regulation. These findings underscore the importance of the miR-876-SOCS4-STAT3 axis in OSCC progression, recurrence, and immune evasion.

Previous research by Ganci et al. identified a recurrence prediction signature for OSCC based on the upregulation of four microRNAs—miR-96-5p, miR-21-3p, miR-141-3p, and miR-130b-3p—when comparing tumors to normal counterparts [31]. However, since both the tumor and adjacent normal tissue in the oral cavity are exposed to the same environmental carcinogens, differential expression analysis might overlook the true contribution of these environmental factors. MicroRNAs not only play a critical role in cellular regulation but also offer high stability and are easily detectable, making them valuable tools for monitoring disease progression [32]. Moreover, microRNA oligonucleotides can effectively inhibit antimiR function [33] as demonstrated in therapies like anti-miR-93-5p, which has shown promise in improving sepsis survival [34]. Given that local recurrent oral cancer often presents as micrometastasis or superficial lesions, which can be treated with photoimmunotherapy [35], targeting microRNAs therapeutically could be an effective strategy. Delivery methods such as lipid nanoparticles (LNPs) or other nanocarriers could facilitate the use of miR-876 as a therapeutic target to prevent OSCC progression and recurrence [36, 37]. Our findings may provide a novel candidate for oral cancer recurrent monitoring and therapeutical niches to prevent disease progression.

Platinum-based chemotherapy remains the standard treatment for OSCC. Recent research by Kang et al. demonstrated that miR-876 in cancer-associated fibroblast-derived extracellular vesicles contributes to OSCC resistance to cisplatin [38]. Their findings also showed that antimir-mediated inhibition of miR-876-3p can restore cisplatin efficacy, which aligns with our results regarding miR-876’s role in chemoresistance. Furthermore, our study extends the understanding of miR-876’s involvement in the SOCS4-STAT3-PD-L1 axis, particularly in adaptive immune evasion. This mechanism has the potential to transform immune “cold” tumors into “hot” tumors, thereby enhancing the effectiveness of immune checkpoint therapies [39, 40].

STAT3 activation plays a pivotal role in the progression and maintenance of oral squamous cell carcinoma (OSCC), primarily by enhancing tumor cell survival, immune evasion, and drug resistance [41]. STAT3 is frequently overexpressed and constitutively activated in OSCC, driving tumor growth and stemness through the regulation of genes associated with self-renewal, epithelial-to-mesenchymal transition (EMT) [42], and the suppression of anti-tumor immune responses [19]. In cancer stem cells (CSCs), STAT3 gain-of-function mutations or persistent activation further amplify these effects by promoting the expression of stemness-related transcription factors such as NANOG, SOX2, and OCT4, which are critical for maintaining the CSC phenotype [43]. This persistent activation is often driven by upstream signaling pathways involving interleukin-6 (IL-6) and epidermal growth factor receptor (EGFR), which promote STAT3 phosphorylation and nuclear translocation [44]. By sustaining a stem-like phenotype and contributing to chemoresistance, STAT3 serves as a critical oncogenic node in OSCC [45]. Importantly, pharmacological inhibition of STAT3 has demonstrated the ability to suppress OSCC growth and diminish cancer stem cell characteristics, underscoring its potential as a therapeutic target to improve treatment outcomes and overcome resistance mechanisms in OSCC [46].

Interestingly, smoking has been associated with varying outcomes in cancer patients. Lieke et al. reported that oral cancer patients who continue smoking have a 21–35% lower 5-year survival rate compared to those who quit smoking [47]. However, some prior meta-analyses suggest that smokers treated with immunotherapy may experience improved treatment outcomes compared to non-smokers [48, 49], potentially due to a higher tumor mutation burden [50]. may be due to high tumor mutation burden Our study provides further insight into this by revealing that NNK, a tobacco-specific carcinogen, induces PD-L1 expression through the SOCS4-STAT3 axis. This finding suggests that NNK contributes to the enhancement of the tumor microenvironment’s inflammatory response and immune evasion, offering a novel mechanism that could be targeted to improve outcomes in OSCC patients.

## Conclusion

Our study identifies miR-876-5p as a key regulator of OSCC progression, recurrence, chemoresistance, and immune evasion through its effects on the SOCS4-STAT3 axis and PD-L1 expression. These findings suggest that miR-876-5p could serve as both a prognostic biomarker and a therapeutic target in OSCC. Future research should focus on developing miR-876-5p inhibitors or modulators as potential therapeutic agents, as well as exploring the use of combination therapies that target both miR-876 and immune checkpoints like PD-L1 to overcome chemoresistance and enhance immune response in OSCC patients. Additionally, further studies are needed to investigate the role of environmental factors, such as NNK, in miR-876 regulation, which may provide insights into prevention strategies for OSCC.

## Electronic supplementary material

Below is the link to the electronic supplementary material.

Supplementary Material 1

Supplementary Material 2

Supplementary Material 3

Supplementary Material 4

Supplementary Material 5

## Data Availability

No datasets were generated or analysed during the current study.
