# Supplementary material for: The miR-876-5p/SOCS4/STAT3 pathway induced the expression of PD-L1 and suppressed antitumor immune responses
Source: Cancer Cell Int. 2025 Mar 26;25:114. doi: 10.1186/s12935-025-03704-2 (PMC11938556; doi:10.1186/s12935-025-03704-2)
Supplement: Supplementary file 1 — Supplementary Material 1 [file 12935_2025_3704_MOESM1_ESM.docx]

Figure S1. Epigenetic regulation of miR-876/873 cluster. 5-AZA treatment restore cluster miR-876-5p and miR-873-5p expression. U6 small nuclear RNA was used as the internal normalization control.
