# Supplementary material for: The miR-876-5p/SOCS4/STAT3 pathway induced the expression of PD-L1 and suppressed antitumor immune responses
Source: Cancer Cell Int. 2025 Mar 26;25:114. doi: 10.1186/s12935-025-03704-2 (PMC11938556; doi:10.1186/s12935-025-03704-2)
Supplement: Supplementary file 2 — Supplementary Material 2 [file 12935_2025_3704_MOESM2_ESM.docx]

Table S1 **Cell line background**

| Cell Name | Cell bank | Catalogue Number | RRID |
| --- | --- | --- | --- |
| Jurkat | BCRC | 60424 | CVCL_0065 |
| 293T | ATCC | CRL-3216 | CVCL_0063 |
| SCC-4 | ATCC | CRL-1624 | CVCL_1684 |
| SCC-9 | ATCC | CRL-1629 | CVCL_1685 |
| SCC-15 | ATCC | CRL-1623 | CVCL_1681 |
| SCC-25 | ATCC | CRL-1628 | CVCL_1681 |
| Cal-27 | ATCC | CRL-2095 | CVCL_1107 |
| Cal-33 | DSMZ | ACC 447 | CVCL_1108 |
| OEC-M1 | NDMC |  | CVCL_6782 |
| HSC-2 | JCRB | JCRB0622 | CVCL_1287 |
| HSC-3 | JCRB | JCRB0623 | CVCL_1288 |
| HSC-3-M3 | JCRB | JCRB1354 | CVCL_8323 |
| HSC-4 | JCRB | JCRB0624 | CVCL_1289 |
| Ca9-22 | JCRB | JCRB0625 | CVCL_1102 |
| OSC-19 | JCRB | JCRB0198 | CVCL_3086 |
| OSC-20 | JCRB | JCRB0197 | CVCL_3087 |
| SAS | JCRB | JCRB0620 | CVCL_1675 |

**Primers**

| human | SOCS4 Forward Primer | NM_199421.2 | GGGTAAGCACAGACTTGTCTCAG |
| --- | --- | --- | --- |
| human | SOCS4 Primer Reverse Primer | NM_199421.2 | TCACAGAGCCAGTCATAGGACC |
| human | NANOG Forward Primer | NM_024865 | CTCCAACATCCTGAACCTCAGC |
| human | NANOG Reverse Primer | NM_024865 | CGTCACACCATTGCTATTCTTCG |
| human | NOTCH1 Forward Primer | NM_017617 | GGTGAACTGCTCTGAGGAGATC |
| human | NOTCH1 Reverse Primer | NM_017617 | GGATTGCAGTCGTCCACGTTGA |
| human | Oct4 (POU5F1) Forward Primer | NM_002701 | CCTGAAGCAGAAGAGGATCACC |
| human | Oct4 (POU5F1) Reverse Primer | NM_002701 | AAAGCGGCAGATGGTCGTTTGG |
| human | SOX2 Forward Primer | NM_003106 | GCTACAGCATGATGCAGGACCA |
| human | SOX2 Reverse Primer | NM_003106 | TCTGCGAGCTGGTCATGGAGTT |
| human | ABCG2 Forward Primer | NM_004827 | GTTCTCAGCAGCTCTTCGGCTT |
| human | ABCG2 Reverse Primer | NM_004827 | TCCTCCAGACACACCACGGATA |
| human | PCNA Forward Primer | NM_002592 | CAAGTAATGTCGATAAAGAGGAGG |
| human | PCNA Reverse Primer | NM_002592 | GTGTCACCGTTGAAGAGAGTGG |
| human | Ki67 (MKI67) Forward Primer | NM_002417 | GAAAGAGTGGCAACCTGCCTTC |
| human | Ki67 (MKI67) Reverse Primer | NM_002417 | GCACCAAGTTTTACTACATCTGCC |
| human | PDL-1 Forward Primer | NM_014143 | TGC CGA CTA CAA GCG AAT TAC TG |
| human | PDL-1 Primer Reverse Primer | NM_014143 | CTG CTT GTC CAG ATG ACT TCG G |
| human | GAPDH Forward Primer | NM_002046 | GTCTCCTCTGACTTCAACAGCG |
| human | GAPDH Reverse Primer | NM_002046 | ACCACCCTGTTGCTGTAGCCAA |

**Antibody list**

| Gene name | Brand | Catalogue Number |
| --- | --- | --- |
| Notch1 | Cell Signaling | 3608 |
| SOX2 | Cell Signaling | 3579 |
| Oct4 | Cell Signaling | 2750 |
| phosphor-STAT3 | Cell Signaling | 9145 |
| STAT3 | BD Biosciences | 610189 |
| GAPDH | GeneTex | GTX100118 |
| β-actin | Abclonal | AC026 |
| Nanog | Proteintech | 14295-1-AP |
| PD-L1 | Proteintech | 17952-1-AP |
| CD25-PE | BD Biosciences | 341009 |
| CD69-PE | BD Biosciences | 341652 |
| CD274 (PD-L1)-APC | Biolegend | 329707 |
| CD45-APC | Biolegend | 368511 |
| CD69-FITC | Biolegend | 310903 |

| **Chemical reagents** | | |
| --- | --- | --- |
| **Name** | **Brand** | **Catalogue number** |
| TransIT-X2 | Mirus | MIR 6000 |
| Blasticidin | Invivogen | ant-bl-05 |
| Fibronectin | Sigma | F1141 |
| GeneArt™ site-directed mutagenesis system | Invitrogen | A13282 |
| HE swift cloning kit | Biotools Co. | TB-VTT-BB05 |
| Hoechst 33342 | Invitrogen | H3570 |
| Human IL12 ELISA kit | R&D | D1200 |
| Immobilon western chemiluminescent HRP substrate | Millipore | WBKLS0500 |
| Lipofectamine 2000 | Thermo | 11668030 |
| LR Clonase II plus enzyme | Invitrogen | 12538-200 |
| ChamQ Universal MasterMix | Vazyme | Q711 |
| ONE-Glo™ Luciferase assay system | Promega | E6110 |
| Pierce™ BCA Protein Assay Kit | Thermo | 23225 |
| Puromycin | Invivogen | ant-pr-1 |
| NxtScript Reverse Transcriptase | Roche | 07051166103 |
| TRIzol™ Reagent | Invitrogen | 15596026 |
| miScript II RT Kit | QIAGEN | 218160 |
| anti-CD3 antibody (Clone:OKT3) | BioLegend | 317353 |
| anti-CD28 antibody (Clone: CD28.2) | BioLegend | 302977100 |
| IL-12 | R&D | 219-IL |
| CCK8 Kit | dojindo | CK04 |
| miR-876 | Genomics |  |
| miR-NC | Genomics |  |
| jetPEI | PolyPlus | 101000053 |

**Vectors**

| **Name** | **Brand** | **Catalogue number** |
| --- | --- | --- |
| 7TFP-CDH1 reporter | Addgene | 91704 |
| pGL3 2 kb prom. CD274 | Addgene | 107003 |
| pGreenFire1-NOTCH1 | SBI | TR020PA |
| pGreenFire1-Nanog | SBI | TR019PA |
| pGreenFire-OCT4 | SBI | TR039PA |
| pGIPZ-miR-876 | HORIZON DISCOVERY |  |
| pGIPZ-Empty | HORIZON DISCOVERY |  |
| pDONR223_STAT3_WT | Addgene | 82235 |
| pDONR221-SOCS4 | DNASU | HsCD00442561 |
| pLenti6.3-DEST | Invitrogen | V53306 |
| pGreenFire-CMV-CpoI | SBI | TR011PA-1 |
