# Supplementary material for: The miR-876-5p/SOCS4/STAT3 pathway induced the expression of PD-L1 and suppressed antitumor immune responses
Source: Cancer Cell Int. 2025 Mar 26;25:114. doi: 10.1186/s12935-025-03704-2 (PMC11938556; doi:10.1186/s12935-025-03704-2)
Supplement: Supplementary file 3 — Supplementary Material 3 [file 12935_2025_3704_MOESM3_ESM.docx]

**Table S2. Relative Protein Expression Quantification from All Western Blot Analysis in the** **manuscript**

| **Figure** | **Cell line** | **Target Protein** | **miR-NS** | **miR-876** | **Significant** |
| --- | --- | --- | --- | --- | --- |
| **2J** | Cal-27 | SOX2 | 0.771±0.010 | 1.712±0.072 | **** |
|  |  | NOTCH1 | 0.853±0.030 | 1.193±0.021 | *** |
|  |  | NANOG | 1.032±0.004 | 1.381±0.024 | **** |
|  |  | OCT4 | 0.505±0.003 | 0.657±0.005 | **** |
|  | SAS | SOX2 | 0.419±0.012 | 0.528±0.006 | *** |
|  |  | NOTCH1 | 0.262±0.005 | 0.333±0.000 | **** |
|  |  | NANOG | 0.215±0.003 | 0.268±0.009 | ** |
|  |  | OCT4 | 0.418±0.006 | 0.536±0.020 | ** |

| **Figure** | **Cell line** | **Target Protein** | **Sponge EV** | **miR-876-SP** | **Significant** |
| --- | --- | --- | --- | --- | --- |
| **2K** | OSC-19 | SOX2 | 0.885±0.039 | 0.666±0.035 | ** |
|  |  | NOTCH1 | 0.800±0.032 | 0.521±0.028 | ** |
|  |  | NANOG | 0.500±0.024 | 0.379±0.021 | * |
|  |  | OCT4 | 1.162±0.017 | 0.857±0.013 | ** |

| **Figure** | **Cell line** | **Target Protein** | **miR-NS** | **miR-876** | **Significant** |
| --- | --- | --- | --- | --- | --- |
| **3E** | Cal-27 | SOCS4 | 0.413±0.026 | 0.304±0.019 | * |
|  | SCC-4 | SOCS4 | 0.594±0.006 | 0.361±0.006 | **** |
|  | SCC-15 | SOCS4 | 1.435±0.005 | 1.067±0.024 | **** |

| **Figure** | **Cell line** | **Target Protein** | **shLacZ** | **shSOCS4** | **Significant** |
| --- | --- | --- | --- | --- | --- |
| **4A** | SCC-4 | SOCS4 | 0.744±0.038 | 0.263±0.015 | **** |
|  |  | STAT3 | 0.558±0.027 | 0.567±0.032 | ns |
|  |  | pSTAT3 | 0.458±0.037 | 0.873±0.041 | *** |
|  | SCC-15 | SOCS4 | 0.293±0.000 | 0.155±0.004 | **** |
|  |  | STAT3 | 0.402±0.004 | 0.351±0.016 | * |
|  |  | pSTAT3 | 0.090±0.010 | 0.365±0.016 | **** |

| **Figure** | | **Cell line** | | | **Target Protein** | | | **oeLacZ** | | | **oeSOCS4** | | | | | **Significant** | | |  |  |
| --- | --- | --- | --- | --- | --- | --- | --- | --- | --- | --- | --- | --- | --- | --- | --- | --- | --- | --- | --- | --- |
| **4B** | | SCC-4 | | | SOCS4 | | | 0.684±0.001 | | | 1.102±0.010 | | | | | **** | | |  |  |
|  |  |  |  |  | STAT3 | | | 0.976±0.011 | | | 0.417±0.004 | | | | | **** | | |  |  |
|  |  |  |  |  | pSTAT3 | | | 0.098±0.002 | | | 0.056±0.008 | | | | | ** | | |  |  |
|  |  | SCC-15 | | | SOCS4 | | | 0.460±0.006 | | | 0.553±0.046 | | | | | * | | |  |  |
|  |  |  |  |  | STAT3 | | | 0.279±0.001 | | | 0.252±0.020 | | | | | ns | | |  |  |
|  |  |  |  |  | pSTAT3 | | | 0.956±0.048 | | | 0.469±0.056 | | | | | *** | | |  |  |
| **Figure** | | **Cell line** | | **Target Protein** | | | **LacZ+ miR-NC** | | | **LacZ+ miR-876** | | | **Significant** | | **oeSOCS4+ miR-876** | | | **Significant** | | |
| **5C** | SCC-4 | | SOCS4 | | | 1.122±0.052 | | | 0.713±0.014 | | | **** | | 1.064±0.105 | | | ns | | |  |
|  |  |  | STAT3 | | | 0.928±0.023 | | | 1.418±0.147 | | | *** | | 1.071±0.145 | | | ns | | |  |
|  |  |  | pSTAT3 | | | 1.079±0.007 | | | 1.544±0.194 | | | * | | 1.099±0.129 | | | ns | | |  |
|  | SCC-15 | | SOCS4 | | | 1.217±0.032 | | | 0.653±0.044 | | | *** | | 1.092±0.224 | | | ns | | |  |
|  |  |  | STAT3 | | | 1.293±0.047 | | | 1.800±0.016 | | | *** | | 1.223±0.046 | | | ns | | |  |
|  |  |  | pSTAT3 | | | 1.288±0.045 | | | 1.785±0.243 | | | * | | 1.231±0.047 | | | ns | | |  |

| **Figure** | **Cell line** | **Target Protein** | **oeLacZ** | **oeSTAT3** | **Significant** |
| --- | --- | --- | --- | --- | --- |
| **6A** | Cal-27 | STAT3 | 0.908±0.000 | 0.955±0.016 | * |
|  |  | pSTAT3 | 0.799±0.002 | 1.142±0.007 | **** |
|  |  | PD-L1 | 0.515±0.021 | 0.759±0.017 | *** |
|  | SCC-4 | STAT3 | 0.249±0.012 | 1.162±0.018 | **** |
|  |  | pSTAT3 | 0.074±0.007 | 0.204±0.007 | **** |
|  |  | PD-L1 | 2.057±0.061 | 2.610±0.205 | * |
|  | SCC-15 | STAT3 | 0.490±0.009 | 1.510±0.066 | **** |
|  |  | pSTAT3 | 0.029±0.002 | 0.443±0.029 | **** |
|  |  | PD-L1 | 0.678±0.014 | 0.927±0.052 | ** |

| **Figure** | **Cell line** | **Target Protein** | **siNS** | **siSTAT3** | **Significant** |
| --- | --- | --- | --- | --- | --- |
| **6B** | Cal-27 | STAT3 | 1.400±0.056 | 0.938±0.076 | ** |
|  |  | pSTAT3 | 1.024±0.027 | 0.896±0.055 | * |
|  |  | PD-L1 | 1.129±0.024 | 0.963±0.010 | *** |
|  | SCC-4 | STAT3 | 1.279±0.032 | 0.393±0.021 | **** |
|  |  | pSTAT3 | 0.348±0.018 | 0.211±0.021 | ** |
|  |  | PD-L1 | 0.712±0.011 | 0.515±0.009 | **** |
|  | SCC-15 | STAT3 | 1.002±0.015 | 0.114±0.008 | **** |
|  |  | pSTAT3 | 1.922±0.117 | 1.242±0.094 | ** |
|  |  | PD-L1 | 2.061±0.088 | 1.085±0.059 | *** |

| **Figure** | **Cell line** | **Target Protein** | **DMSO** | **25 mM NNK** | **Significant** | **50 mM NNK** | **Significant** |
| --- | --- | --- | --- | --- | --- | --- | --- |
| **7C** | SCC-4 | SOCS4 | 0.530±0.002 | 0.422±0.013 | *** | 0.174±0.002 | **** |
|  | Cal-27 | SOCS4 | 0.869±0.006 | 0.868±0.026 | ns | 0.508±0.011 | **** |

| **Figure** | **Cell line** | **Target Protein** | **DMSO** | **25 mM NNK** | **Significant** | **50 mM NNK** | **Significant** |
| --- | --- | --- | --- | --- | --- | --- | --- |
| **7D** | SCC-4 | STAT3 | 0.334±0.004 | 0.313±0.005 | * | 0.452±0.009 | **** |
|  |  | pSTAT3 | 1.653±0.076 | 2.148±0.080 | ** | 2.806±0.127 | *** |
|  |  | PD-L1 | 0.440±0.003 | 0.568±0.016 | *** | 0.422±0.002 | ** |
|  | Cal-27 | STAT3 | 0.381±0.010 | 0.543±0.007 | **** | 0.427±0.008 | ** |
|  |  | pSTAT3 | 1.339±0.025 | 1.876±0.247 | * | 1.924±0.248 | * |
|  |  | PD-L1 | 0.171±0.004 | 0.489±0.025 | **** | 0.542±0.005 | **** |

ns: non-significant; *: P <0.05; **: P <0.01; ***: P <0.001; ****: P <0.0001
