# Supplementary material for: The miR-876-5p/SOCS4/STAT3 pathway induced the expression of PD-L1 and suppressed antitumor immune responses
Source: Cancer Cell Int. 2025 Mar 26;25:114. doi: 10.1186/s12935-025-03704-2 (PMC11938556; doi:10.1186/s12935-025-03704-2)
Supplement: Supplementary file 4 — Supplementary Material 4 [file 12935_2025_3704_MOESM4_ESM.docx]

| **Table S3. List of Recurrent microRNA** | | | | | | |
| --- | --- | --- | --- | --- | --- | --- |
| **TargetID** | **Average Recurrent** | **StD Recurrent** | **Average Non- Recurrent** | **StD Non- Recurrent** | **Ratio** | **P Value** |
| hsa-miR-876-5p | 171.09 | 116.88 | 81.25 | 23.28 | 2.11 | 0.0002 |
| hsa-miR-219-1-3p | 81.74 | 107.29 | 42.09 | 11.12 | 1.94 | 0.0479 |
| hsa-miR-644 | 84.12 | 49.15 | 43.75 | 8.44 | 1.92 | 0.0001 |
| hsa-miR-612 | 387.40 | 409.20 | 208.46 | 134.38 | 1.86 | 0.0406 |
| hsa-miR-1268 | 959.04 | 467.31 | 580.42 | 327.04 | 1.65 | 0.0072 |
| hsa-miR-708* | 131.55 | 69.41 | 80.65 | 37.91 | 1.63 | 0.0055 |
| hsa-miR-584 | 534.00 | 335.23 | 329.26 | 206.46 | 1.62 | 0.0267 |
| hsa-miR-1300 | 311.91 | 124.28 | 197.15 | 111.24 | 1.58 | 0.0092 |
| hsa-miR-503 | 2915.24 | 1545.19 | 1883.18 | 847.94 | 1.55 | 0.0109 |
| hsa-miR-363 | 387.49 | 158.17 | 251.69 | 114.86 | 1.54 | 0.0056 |
| hsa-miR-1244 | 113.53 | 71.70 | 74.29 | 36.13 | 1.53 | 0.0280 |
| hsa-miR-1468 | 44.89 | 35.41 | 29.79 | 1.56 | 1.51 | 0.0218 |
| hsa-miR-661 | 1498.11 | 473.75 | 1038.80 | 491.59 | 1.44 | 0.0138 |
| hsa-miR-337:9.1 | 75.12 | 29.35 | 52.33 | 23.78 | 1.44 | 0.0178 |
| hsa-miR-1228* | 4095.88 | 1617.86 | 2879.96 | 1325.01 | 1.42 | 0.0225 |
| hsa-miR-1250 | 78.15 | 29.40 | 56.83 | 17.55 | 1.38 | 0.0083 |
| hsa-miR-638 | 62.53 | 34.46 | 45.66 | 16.46 | 1.37 | 0.0432 |
| hsa-miR-130b | 892.83 | 342.59 | 662.44 | 226.25 | 1.35 | 0.0195 |
| hsa-miR-22* | 3380.36 | 1327.79 | 2526.33 | 941.98 | 1.34 | 0.0313 |
| hsa-miR-324-5p | 1992.48 | 578.19 | 1511.65 | 511.60 | 1.32 | 0.0171 |
| hsa-miR-744* | 79.39 | 38.18 | 61.22 | 17.69 | 1.30 | 0.0464 |
| hsa-miR-181b | 2920.71 | 695.82 | 2258.02 | 756.27 | 1.29 | 0.0193 |
| hsa-miR-1255a | 43.13 | 22.01 | 33.66 | 6.89 | 1.28 | 0.0415 |
| hsa-miR-1301 | 280.36 | 94.36 | 225.94 | 56.25 | 1.24 | 0.0328 |
| hsa-miR-1308 | 22440.09 | 3908.45 | 18383.31 | 4596.41 | 1.22 | 0.0168 |
| hsa-miR-1224-5p | 42.52 | 17.67 | 35.15 | 5.66 | 1.21 | 0.0489 |
| hsa-miR-193b | 9676.07 | 2926.25 | 8015.68 | 1875.13 | 1.21 | 0.0429 |
| hsa-miR-552 | 47.27 | 17.79 | 39.54 | 5.34 | 1.20 | 0.0376 |
| hsa-miR-518e | 54.54 | 12.09 | 46.71 | 5.06 | 1.17 | 0.0059 |
| hsa-miR-95 | 13301.26 | 2271.17 | 11491.95 | 2434.40 | 1.16 | 0.0456 |
| hsa-miR-15b | 9664.13 | 1178.00 | 8442.75 | 1585.58 | 1.14 | 0.0316 |
| hsa-miR-497* | 32.82 | 8.27 | 28.82 | 1.13 | 1.14 | 0.0120 |
| hsa-miR-555 | 30.67 | 0.97 | 31.45 | 1.03 | 0.98 | 0.0428 |
| hsa-miR-1256 | 41.89 | 2.31 | 45.42 | 5.01 | 0.92 | 0.0391 |
| hsa-miR-27b | 14748.51 | 1779.77 | 16119.09 | 1666.86 | 0.91 | 0.0328 |
| hsa-miR-548p | 40.33 | 3.34 | 44.49 | 6.08 | 0.91 | 0.0473 |
| hsa-miR-19b-2* | 59.22 | 6.97 | 68.02 | 12.94 | 0.87 | 0.0481 |
| hsa-miR-30e* | 3211.32 | 896.72 | 3793.52 | 542.71 | 0.85 | 0.0179 |
| hsa-miR-148b | 1324.37 | 345.63 | 1647.32 | 443.36 | 0.80 | 0.0429 |
| hsa-miR-148a | 10422.93 | 3309.36 | 13382.78 | 3548.04 | 0.78 | 0.0258 |
| hsa-miR-126* | 6603.51 | 1672.95 | 8679.07 | 1991.90 | 0.76 | 0.0053 |
| hsa-miR-889 | 328.20 | 86.67 | 432.65 | 135.36 | 0.76 | 0.0284 |
| hsa-miR-29c | 5535.62 | 2107.01 | 7466.00 | 2667.80 | 0.74 | 0.0447 |
| hsa-miR-10b | 2639.05 | 1031.86 | 3596.22 | 1158.73 | 0.73 | 0.0258 |
| hsa-miR-544 | 17447.58 | 9588.69 | 24236.79 | 3038.14 | 0.72 | 0.0013 |
| hsa-miR-186 | 1165.85 | 529.24 | 1622.46 | 591.50 | 0.72 | 0.0367 |
| hsa-miR-199a*:9.1 | 8272.67 | 3769.96 | 12015.78 | 3442.08 | 0.69 | 0.0060 |
| hsa-miR-374b* | 57.83 | 17.57 | 89.50 | 47.50 | 0.65 | 0.0476 |
| hsa-miR-944 | 365.78 | 189.59 | 612.49 | 324.30 | 0.60 | 0.0291 |
| hsa-miR-181c | 212.01 | 142.46 | 369.10 | 214.84 | 0.57 | 0.0380 |
| hsa-miR-520e | 31.80 | 2.17 | 128.70 | 137.72 | 0.25 | 0.0335 |
