# Supplementary material for: The miR-876-5p/SOCS4/STAT3 pathway induced the expression of PD-L1 and suppressed antitumor immune responses
Source: Cancer Cell Int. 2025 Mar 26;25:114. doi: 10.1186/s12935-025-03704-2 (PMC11938556; doi:10.1186/s12935-025-03704-2)
Supplement: Supplementary file 5 — Supplementary Material 5 [file 12935_2025_3704_MOESM5_ESM.docx]

| **Table S4. Correlation among miR-876-SOCS4-CD274 axis** | | | | |
| --- | --- | --- | --- | --- |
|  | **hsa-miR-876-5p** | **SOCS4** | **CD274** | **STAT3** |
| **Recurrent 1** | 0.57 | -0.62 | 1.32 | -0.16 |
| **Recurrent 2** | 1.42 | -0.78 | 0.98 | -0.43 |
| **Recurrent 3** | 0.66 | -0.60 | 1.14 | -0.28 |
| **Recurrent 4** | 0.60 | -0.45 | 1.04 | -0.34 |
| **Recurrent 5** | 2.20 | -1.35 | 1.65 | -0.25 |
| **Recurrent 6** | 0.69 | -0.67 | 1.03 | -0.30 |
| **Recurrent 7** | 0.74 | -0.79 | 1.15 | -0.31 |
| **Recurrent 8** | 2.17 | -0.98 | 2.06 | -0.25 |
| **Recurrent 9** | 1.86 | -0.86 | 1.51 | 0.10 |
| **Recurrent 10** | 1.37 | -0.80 | 1.14 | 0.00 |
| **Non-Recurrent 1** | 0.00 | 0.00 | 0.00 | 0.00 |
| **Non-Recurrent 2** | 0.21 | -0.21 | 0.34 | 0.05 |
| **Non-Recurrent 3** | 0.34 | -0.16 | 1.03 | -0.21 |
| **Non-Recurrent 4** | 0.35 | -0.37 | 0.70 | -0.71 |
| **Non-Recurrent 5** | -0.32 | -0.43 | -0.50 | -0.38 |
| **Non-Recurrent 6** | 0.50 | -0.33 | -0.89 | -0.04 |
| **Non-Recurrent 7** | -0.21 | -0.22 | -0.70 | -0.35 |
| **Non-Recurrent 8** | 0.30 | -0.44 | 1.10 | -0.44 |
| **Non-Recurrent 9** | -0.46 | -0.23 | -0.76 | -0.35 |
| **Non-Recurrent 10** | 0.23 | -0.36 | 0.43 | 0.06 |
| **Non-Recurrent 11** | 0.39 | -0.33 | 0.48 | -0.07 |
| **Non-Recurrent 12** | -0.33 | -0.39 | -0.22 | -0.32 |
| **Non-Recurrent 13** | -0.34 | 0.18 | 0.83 | -0.29 |
| **Non-Recurrent 14** | -0.03 | -0.05 | 0.17 | -0.43 |
| **Non-Recurrent 15** | 0.27 | -0.26 | -0.43 | -0.23 |
| **Non-Recurrent 16** | -0.24 | -0.17 | -0.86 | -0.14 |
| **Non-Recurrent 17** | -0.55 | -0.54 | 0.65 | -0.20 |
| **Non-Recurrent 18** | -0.20 | -0.27 | 0.59 | -0.25 |
| **Non-Recurrent 19** | -0.44 | 0.27 | 0.09 | -0.19 |
| **Non-Recurrent 20** | -0.52 | 0.15 | -1.09 | 0.12 |
| **Non-Recurrent 21** | 0.21 | -0.37 | 0.74 | -0.36 |
| **Non-Recurrent 22** | 0.05 | -0.18 | 0.52 | -0.28 |
| **Non-Recurrent 23** | -0.31 | -0.32 | 0.26 | -0.37 |
| **Non-Recurrent 24** | -0.30 | -0.25 | -1.53 | -0.40 |
| **Non-Recurrent 25** | -0.11 | -0.20 | 0.82 | -0.42 |
| **Non-Recurrent 26** | 0.13 | -0.54 | 0.46 | 0.01 |
| **Non-Recurrent 27** | -0.33 | 0.03 | -1.90 | -0.20 |
| **Non-Recurrent 28** | 0.17 | 0.20 | -0.35 | 0.07 |
| **Non-Recurrent 29** | -0.47 | 0.06 | -0.65 | -0.32 |
| **Non-Recurrent 30** | -0.52 | -0.25 | -0.96 | -0.17 |
